# Paravertebral dexmedetomidine as an adjuvant to ropivacaine protects against independent lung injury during one-lung ventilation: a preliminary randomized clinical trial

**DOI:** 10.1186/s12871-018-0532-6

**Published:** 2018-06-15

**Authors:** Wei Zhang, Shanfeng Zhang, Bing Li, Mingyang Sun, Jiaqiang Zhang

**Affiliations:** 1 Department of Anesthesiology, Henan Provincial People’s Hospital, No. 7, Weiwu Road, Zhengzhou City, Henan Province China; 2 Department of Biochemistry and Molecular Biology, The Academy of Medical Science, Zhengzhou University, No. 100, Science Avenue, Zhengzhou City, Henan Province China

**Keywords:** Paravertebral, Dexmedetomidine, Lung injury, One-lung ventilation

## Abstract

**Background:**

To investigate the effect of paravertebral dexmedetomidine as an adjuvant to ropivacaine on independent lung injury during one-lung ventilation.

**Methods:**

In total, 120 patients who underwent elective radical resection of pulmonary carcinoma were randomly assigned to one of six groups (*n* = 20): normal saline (C group), ropivacaine (R group), intravenous dexmedetomidine (Div group), 0.5 μg/kg paravertebral dexmedetomidine as an adjuvant to ropivacaine (RD0.5 group), 1.0 μg/kg paravertebral dexmedetomidine as an adjuvant to ropivacaine (RD1.0 group), or 2.0 μg/kg paravertebral dexmedetomidine as an adjuvant to ropivacaine (RD2.0 group).

Patients in the R, Div, RD0.5, RD1.0 and RD2.0 groups underwent a thoracic paravertebral block, and normal saline was administered as a control to C group. Small marginal lung samples next to the tumor were harvested immediately after the tumor tissues were excised.

Lung injury was evaluated as follows: an injury score was determined via light microscopy, and cell apoptosis was determined via a TUNEL assay. TNF-α, IL-6, miRNA-210, HIF-1α, Tom20 and ISCU2 were also detected.

**Results:**

Both intravenous and paravertebral dexmedetomidine attenuated independent lung injury. Downregulation of HIF-1α and miRNA-210 and upregulation of Tom20 and ISCU2 may be the underlying mechanism. No difference was observed between the Div and RD0.5 groups, and no further improvement of lung injury was found in the RD1.0 and RD2.0 groups with increased paravertebral dexmedetomidine doses.

**Conclusions:**

Paravertebral dexmedetomidine as an adjuvant to ropivacaine, which is comparable to intravenous dexmedetomidine, could protect against independent lung injury during one-lung ventilation.

**Trial registration:**

ISRCTN, 13000406; retrospectively registered on 22.05.2018.

## Background

One-lung ventilation (OLV) has enabled increasingly complex intrathoracic surgery with increased use of minimally invasive techniques. However, OLV is associated with postoperative pulmonary complications in 20% of lung resections [1]. It is well known that before re-inflation, the independent lung must maintain no ventilation and that, thereafter, the oxygen tension in the alveoli decreases [2]. As the duration of collapse increases, independent lung injury becomes increasingly more severe. Reactive oxygen species and edema of the alveolar-capillary membrane are activated in the collapsed lung [3], and neutrophil infiltration and alveolar structural damage increase with the duration of lung collapse [4].

However, no single intervention has been proven to be helpful in reversing the collapsed lung injury. Dexmedetomidine (DEX), an α2 adrenoceptor agonist, has been proven to exert lung protective effects in many in vivo and in vitro lung injury models, including models of one-lung ventilation [5–7]. OLV can cause the production of many pro-inflammatory cytokines and reactive oxygen species, which are adverse factors. The organ protective mechanism of DEX involves inhibition of inflammation.

With the recent introduction of ultrasound-guided techniques, thoracic paravertebral block (TPVB) has become more accessible to anesthesiologists worldwide [8]. As an adjuvant to local anesthesia, DEX is regarded to be effective in preoperative analgesic management [9, 10]. In previous studies, both pro-analgesia and pro-sedation were observed preoperatively when paravertebral DEX was administered. The advantage of paravertebral DEX is that it can induce both a peripheral effect and a systemic effect. Based on these results, we believe that paravertebral DEX could exert not only a local pro-analgesia effect but also the same systemic effect as intravenous administration. Whether or not paravertebral DEX as an adjuvant to local anesthetics can confer lung protection in OLV remains unclear.

Transient bradycardia and hypotension could be found when high dose of peripheral DEX was administered. Previous studies with various doses of peripheral DEX between 20 and 150 μg or up to 2 μg/kg as an additive to local anesthetics, have been published [11, 12]. The optimal dose of paravertebral DEX has yet to be identified. Based on previous studies [13, 14] and our pilot experiment, three doses (0.5 μg/kg, 1 μg/kg, 2 μg/kg) of paravertebral DEX were discussed in our study.

We hypothesized that paravertebral DEX would alleviate independent lung injury as effectively as intravenously administered DEX. The primary outcome is the lung injury score, the second outcome includes the hemodynamics, apoptosis, inflammatory cytokines. To further elucidate the mechanism, some indexes related to hypoxia and mitochondrial injury were also evaluated.

## Methods

### Study design and setting

This randomized, double-blind study enrolled 133 patients from June 2016 to March 2017. This prospective study was approved by the Ethics Committee of Henan Provincial People’s Hospital, and written informed consent was obtained from the patients. The study protocol complied with the 1975 Declaration of Helsinki.

### Characteristics of participants

Patients who were classified as American Society of Anesthesiologists (ASA) physician status I or II, aged 18–65 years, and scheduled for selective radical resection of pulmonary carcinoma were enrolled. Patients with hypertension, diabetes mellitus, heart diseases, suggestive history of inflammation or coagulation dysfunction were excluded.

### Preoperative preparations and anesthesia protocol

No patients received pretreatment before admission to the operating room. All patients were monitored by electrocardiography, pulse oximetry, invasive blood pressure recordings and bispectral index values. For fluid supplementation, 3–5 ml/kg/h crystalloid was administered.

After written informed consent was obtained, all patients were randomly assigned (by a random digital table generated by a computer) to one of six groups (*n* = 20): normal saline (C group), ropivacaine (R group), intravenous DEX (Div group), 0.5 μg/kg paravertebral DEX as an adjuvant to ropivacaine (RD0.5 group), 1.0 μg/kg DEX as an adjuvant to ropivacaine (RD1.0 group), and 2.0 μg/kg paravertebral DEX as an adjuvant to ropivacaine (RD2.0 group).

In this study, each patient had two surgical incisions. The pain conduction of each surgical incision is dictated by the intercostal nerve, which come from the corresponding thoracic paravertebral space respectively. To alleviate the pain, we injected local anesthetic drugs to the corresponding paravertebral space and thus each patient needed a total of two thoracic paravertebral blocks. TPVB was induced under ultrasound guidance (S-Nerve, Fujifilm Sonosite, Inc). TPVB was not administered in the C or Div groups. Patients in the C group received only normal saline as a control; patients in the R group received a total volume of 20 ml 0.5% ropivacaine for the TPVB (cat No. NATM, AstraZeneca, Sweden), with 10 ml injected at each puncture point; patients in the Div group received a total dosage of 0.5 μg/kg intravenous DEX (cat No. 20160301, Jiangsu Nhwa Pharmaceutical Co., Ltd., China) over 10 min; and patients in the RD0.5, RD1.0 and RD2.0 groups received ropivacaine (final concentration of 0.5%) mixed with 0.5 μg/kg, 1.0 μg/kg and 2.0 μg/kg DEX, respectively, as an adjuvant. Only one anesthesiologist who was blinded to the group allocation was involved in the preoperative management.

Anesthesia was induced after the TPVB was completed. Etomidate, sufentanil and rocuronium were intravenously administered for anesthesia induction. A double lumen endotracheal tube was successfully inserted, and the location confirmed with a fiber bronchoscope. Mechanical ventilation (volume-controlled ventilation) was performed after tracheal intubation with a tidal volume of 8 ml/kg predicted body weight (PBW) during two-lung ventilation (TLV) and 6 ml/kg PBW during OLV. The inspired oxygen fraction (F_I_O_2_) was set to 0.5 during TLV and 1.0 during OLV. Respiratory rate was set to 12 breaths/min and adjusted to maintain P_ET_CO_2_ between 35 and 45 mmHg. Positive end-expiratory pressure was maintained at 5 cmH_2_O. Anesthesia was maintained with propofol, remifentanil and cisatracurium. The depth of anesthesia was maintained between BIS 40 and 60 (Aspect, USA) by changing the infusion rate of propofol.

The heart rate (HR), mean arterial pressure, SpO_2_, and arterial oxygen saturation were also routinely monitored at the following time points: (1) preoperative (Pre), 15 min after admission to the operating room without any drug treatment, (2) TLV, immediately before initiation of OLV, (3) OLV_1_, 15 min after initiation of OLV, (4) OLV_2_, 30 min after initiation of OLV, and (5) OLV_3_, immediately before the tumor tissue was excised. Hypoxemia was defined as SpO_2_<90% at any time; bradycardia was defined as HR<55 beats/min; hypotension was defined as MAP<55 mmHg. Atropine (0.5 mg) or methoxamine (1–2 mg), respectively, were administered when bradycardia or hypotension occurred.

### Measurements

The independent lung was kept collapsed during OLV. Immediately after the tumor tissue was excised, small marginal lung samples of approximately 0.5 cm × 0.5 cm × 0.5 cm next to the tumor were harvested as quickly as possible. After gently flushing with PBS, the samples were cut into halves; one part was rapidly transported to a liquid nitrogen canister, and the other part was perfused with 4% formaldehyde.

### Lung injury score by light microscopy

The formaldehyde-infused lung tissues were embedded in paraffin wax, sectioned (5 μm), and then stained with hematoxylin and eosin. The histological changes of the fixed lung tissue were evaluated by an independent pathologist who was blinded to the study protocols. The severity of lung injury was quantified using the 4-point scoring system reported by Kozian [15] et al., which included pulmonary interstitial edema, alveolar edema, alveolar congestion and neutrophil infiltration. Scoring standards were as follows: 0, no change or very mild changes; 1, mild changes; 2, moderate changes; and 3, severe changes. The average lung injury score from three adjacent slices was evaluated. The summation of four scores was recognized as the final lung injury score.

### Detection of cell apoptosis by TUNEL assays

A TUNEL assay was employed according to the manufacturer’s protocol of an in situ cell death detection kit-POD (cat no. 11684817910; Roche, Basel, Switzerland). Apoptotic cells were indicated by brown-yellow granules in the cytoplasm. The number of apoptotic cells in random fields of view (magnification, × 400) was calculated. The apoptosis index (AI; %) was expressed as follows: the number of apoptotic cells/the total number of cells × 100.

### Enzyme-linked immunosorbent assay (ELISA) of inflammatory cytokines

Lung tissues were prepared as 10% tissue homogenates and centrifuged at 3000 rpm at 4 °C for 10 min. The supernatant was collected for further analysis. The concentrations of TNF-α and IL-6 in lung tissues were measured according to the manufacturer’s instructions using ELISA kits (Nanjing Keygen Biotech Co., Ltd.). Absorbance at 450 nm (OD 450) was determined using a microplate reader.

### Real-time PCR of miRNA-210

Using TRIzol reagent (Cat. No. 15596–026, Transgene, France), total RNA of treated lung tissue was isolated to determine the expression of miRNA-210. Relative quantitative PCR (RT-qPCR) and SYBR Green random mixing methods were used to evaluate the mRNA levels of apoptosis-related genes. Glyceraldehyde-3-phosphate dehydrogenase (GAPDH) was used as an internal control. The 2 − ΔΔCt method was used to calculate relative changes in gene expression. The following primers for miRNA-210 were used for quantitative PCR: sense, 5’-CTGTGCGTGTGACAG-3′, and antisense, 5’-GTGCAGGGTCCGAGGT-3′. RT-qPCR was performed following the instructions of the TransStart Top Green qPCR SuperMix kit (TransGen Biotech, Beijing, China). The reaction mixtures were incubated for 30 min at 48 °C, followed by 40 cycles of PCR at 94 °C for 5 s, 55 °C for 15 s, and 72 °C for 10 s. At the end of 40 cycles, a melting curve analysis was performed to confirm the presence of only a single amplified product of the expected size.

### Expression of HIF-1α, Tom20, and ISCU2 in the lung tissues by western blot

Lung tissues obtained from each group were mixed with cracking liquid and centrifuged at 10,000×g at 4 °C. Proteins in the liquid supernatant were quantitated via the bicinchoninic acid method. SDS-PAGE protein electrophoresis was performed, and proteins were transferred to a nitrocellulose membrane. Following transfer to nitrocellulose membranes, the samples were blocked by 5% skim milk powder in Tris-buffered saline with Tween for 1 h at room temperature and then incubated overnight at 4 °C with rabbit polyclonal antibodies against hypoxia inducible factor-1 α (HIF-1α), mitochondrial outer membrane 20 (Tom20) and iron-sulfur cluster assembly enzyme-2 (ISCU2) (Santa Cruz Biotechnology, Inc., Dallas, TX, USA). After washing, samples were incubated for 1 h with rabbit polyclonal antibodies against phosphorylated HIF-1α, Tom and ISCU2; GAPDH rabbit polyclonal antibody was used as a control. Proteins were visualized using enhanced chemiluminescence detection reagents (BeyoECL Plus; cat no. P0018; Beyotime Institute of Biotechnology) and exposed to photographic film for a suitable length of time. The images were analyzed using Bandscan software. The ratios of the gray values of the target protein and β-actin protein stripes provided measurements of the HIF-1α, Tom20 and ISCU2 phosphorylation levels.

### Statistical analysis

The primary objective in the present study is to investigate the effect of paravertebral dexmedetomidine as an adjuvant to ropivacaine on independent lung injury during one-lung ventilation. Based on a previous experiment [16], the mean ± standard deviation for lung injury scores was 4.0 ± 1.4 in the control group, the hypothesis of this study was that there would be a power of 0.8, 20% differences between the control group and the treatment groups in the lung injury scores, with an alpha-error of 0.05 (since the number of pairwise comparisons would be 10, so the alpha-error would be corrected by 10 pairwise comparisons). A sample size of 82 patients was calculated by using “Power and Sample Size.com“, an online power and sample size calculator. Since we only recruit 20 patients in each study arm, the data collected should be regarded as a preliminary analysis.

All data are presented as the mean ± standard deviation. The experimental results were analyzed using SPSS 17.0 software (SPSS, Inc., Chicago, IL, USA). The differences among groups were assessed by one-way analysis of variance with the Bonferroni test for multiple comparisons. A *p* value of less than 0.05 was considered statistically significant.

## Results

**Fig. 1:**
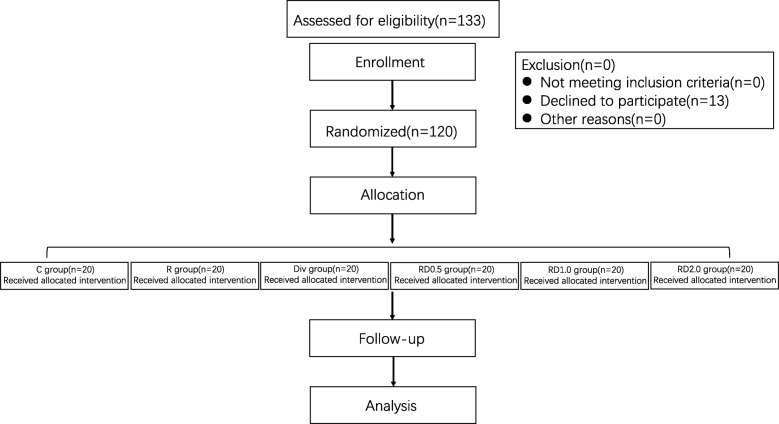
Study flow diagram

In total, 133 patients met the inclusion criteria, of which 13 patients declined to participate. Therefore, a total of 120 patients were enrolled in this study. The final analyses included 120 patients (Fig. 1).

The patients’ baseline characteristics did not differ (Table 1). More patients experienced bradycardia and hypotension in the Div, RD1.0 and RD2.0 groups than in the other three groups. Patients in the RD2.0 group experienced the most bradycardia and hypotension (Table 1).

**Table 1:** Characteristics of the patients

	C Group	R Group	Div Group	RD0.5 Group	RD1.0 Group	RD2.0 Group
Age (year)	49 ± 13	57 ± 15	52 ± 12	56 ± 10	54 ± 13	51 ± 12
Males/females	11/9	10/10	9/11	8/12	12/8	11/9
Height (cm)	158 ± 11	168 ± 11	161 ± 14	165 ± 10	159 ± 12	163 ± 13
Weight (kg)	58 ± 9	65 ± 11	65 ± 12	60 ± 9	57 ± 12	63 ± 9
BMI	21.1 ± 1.8	22.4 ± 2.1	24.1 ± 1.9	21.9 ± 1.7	20.6 ± 2.6	23.1 ± 2.3
ASA I/II	5/15	7/13	5/15	4/16	5/15	4/16
OLV duration (min)	65 ± 14	59 ± 15	61 ± 10	55 ± 9	53 ± 12	63 ± 11
Surgery duration (min)	153 ± 16	149 ± 27	134 ± 23	146 ± 32	143 ± 30	140 ± 25
Infused crystalloid (ml)	644 ± 84	613 ± 88	605 ± 55	599 ± 48	586 ± 55	600 ± 68
Urinary output (ml)	172 ± 18	171 ± 23	175 ± 31	178 ± 36	168 ± 29	164 ± 22
Estimated blood loss (ml)	174 ± 27	172 ± 33	175 ± 31	174 ± 34	165 ± 25	159 ± 21
Bradycardia	0	0	3^ab^	0^c^	3^abd^	6^abcde^
Atropine	0	0	3^ab^	0^c^	3^abd^	6^abcde^
Hypotension	0	0	2 ^ab^	0 ^c^	2 ^abd^	4 ^abcde^
Methoxamine	0	0	2 ^ab^	0 ^c^	2 ^abd^	4 ^abcde^

Values are expressed as the number of patients or the mean ± SD. ASA, American Society of Anesthesiologist Classification. BMI, body mass index. OLV, one-lung ventilation. ^a^*P*<0.05 vs. the C group; ^b^*P*<0.05 vs. the R group; ^c^*P*<0.05 vs. the Div group; ^d^*P*<0.05 vs. the RD0.5 group; ^e^*P*<0.05 vs. the RD1.0 group

The results of intraoperative blood gas and hemodynamics analyses are shown in Table 2. No differences in preoperative PaO_2_, PaCO_2_, SpO_2_, MAP or HR were found among any groups. No hypoxemia was found in any patients. MAP was lower in the Div, RD0.5, RD1.0 and RD2.0 groups at the TLV time point than in the C group. MAP was also lower in the Div, RD0.5, RD1.0 and RD2.0 groups at the OLV_1_ time point than in the C and R groups. HR was lower at the TLV time point in the Div, RD0.5, RD1.0 and RD2.0 groups than in the C and R groups. HR was also lower at the OLV_1_, OLV_2_, and OLV_3_ time points in the Div, RD0.5, RD1.0 and RD2.0 groups than in the C group.

**Table 2:** Intraoperative blood gas and hemodynamics during one-lung ventilation

	C Group	R Group	Div Group	RD0.5 Group	RD1.0 Group	RD2.0 Group
P_a_O_2_(mmHg)						
Pre	83 ± 7	86 ± 8	85 ± 8	87 ± 7	86 ± 7	86 ± 9
TLV	171 ± 24	162 ± 23	158 ± 11	168 ± 21	167 ± 17	164 ± 17
OLV_1_	98 ± 13	95 ± 15	95 ± 10	100 ± 13	96 ± 9	95 ± 11
OLV_2_	91 ± 8	92 ± 6	92 ± 6	92 ± 9	91 ± 7	89 ± 5
OLV_3_	90 ± 6	90 ± 5	91 ± 6	90 ± 6	91 ± 6	89 ± 5
P_a_CO_2_(mmHg)						
Pre	41 ± 3	42 ± 3	41 ± 4	40 ± 4	41 ± 3	41 ± 2
TLV	42 ± 3	42 ± 3	41 ± 4	39 ± 4	41 ± 3	40 ± 3
OLV_1_	44 ± 2	45 ± 2	45 ± 3	44 ± 3	44 ± 2	45 ± 2
OLV_2_	45 ± 3	45 ± 3	44 ± 3	45 ± 2	44 ± 3	44 ± 2
OLV_3_	45 ± 3	45 ± 4	45 ± 4	45 ± 3	43 ± 4	44 ± 4
SpO_2_(%)						
Pre	96.4 ± 1.8	96.1 ± 2.1	96.9 ± 2.1	97.2 ± 1.8	97.2 ± 1.9	96.5 ± 1.9
TLV	98.8 ± 0.9	98.7 ± 1.3	98.8 ± 1.0	99.0 ± 0.8	98.7 ± 1.2	98.8 ± 0.9
OLV_1_	97.2 ± 1.8	97.1 ± 2.1	98.1 ± 1.3	97.5 ± 2.0	97.9 ± 1.6	97.3 ± 1.8
OLV_2_	96.4 ± 1.7	96.8 ± 2.1	96.8 ± 2.2	97.1 ± 1.9	97.3 ± 1.9	96.7 ± 2.0
OLV_3_	95.9 ± 1.7	96.2 ± 2.0	96.4 ± 2.0	96.7 ± 1.8	97.0 ± 1.7	96.6 ± 2.0
MAP(mmHg)						
Pre	82 ± 8	89 ± 11	84 ± 13	87 ± 12	89 ± 11	90 ± 13
TLV	86 ± 7	80 ± 8	72 ± 7^a^	75 ± 7^a^	72 ± 7^a^	74 ± 7^a^
OLV_1_	86 ± 9	80 ± 11	66 ± 6^ab^	68 ± 5^ab^	67 ± 6^ab^	68 ± 7^ab^
OLV_2_	71 ± 7	67 ± 6	65 ± 7	69 ± 5	65 ± 7	65 ± 8
OLV_3_	68 ± 5	66 ± 5	65 ± 7	67 ± 4	63 ± 5	65 ± 7
HR(beats/min)						
Pre	85 ± 6	90 ± 9	87 ± 10	89 ± 10	91 ± 11	91 ± 12
TLV	88 ± 9	90 ± 9	72 ± 9^ab^	75 ± 8^ab^	72 ± 8^ab^	73 ± 8^ab^
OLV_1_	83 ± 9	75 ± 12	68 ± 6^a^	71 ± 7^a^	67 ± 8^a^	69 ± 10^a^
OLV_2_	79 ± 9	72 ± 12	65 ± 6^a^	68 ± 7^a^	67 ± 8^a^	65 ± 11^a^
OLV_3_	78 ± 9	71 ± 11	64 ± 6^a^	67 ± 6^a^	66 ± 6^a^	65 ± 8^a^

TLV: two-lung ventilation (immediately before initiation of OLV); OLV_1_: 15 min of OLV; OLV_2_: 30 min of OLV; OLV_3_: immediately before the tumor tissue was excised; MAP: mean arterial pressure; HR: heart rate. Values are expressed as the mean ± SD. ^a^*P*<0.05 vs. the C group; ^b^*P*<0.05 vs. the R group

### Histological evaluation

**Fig. 2:**
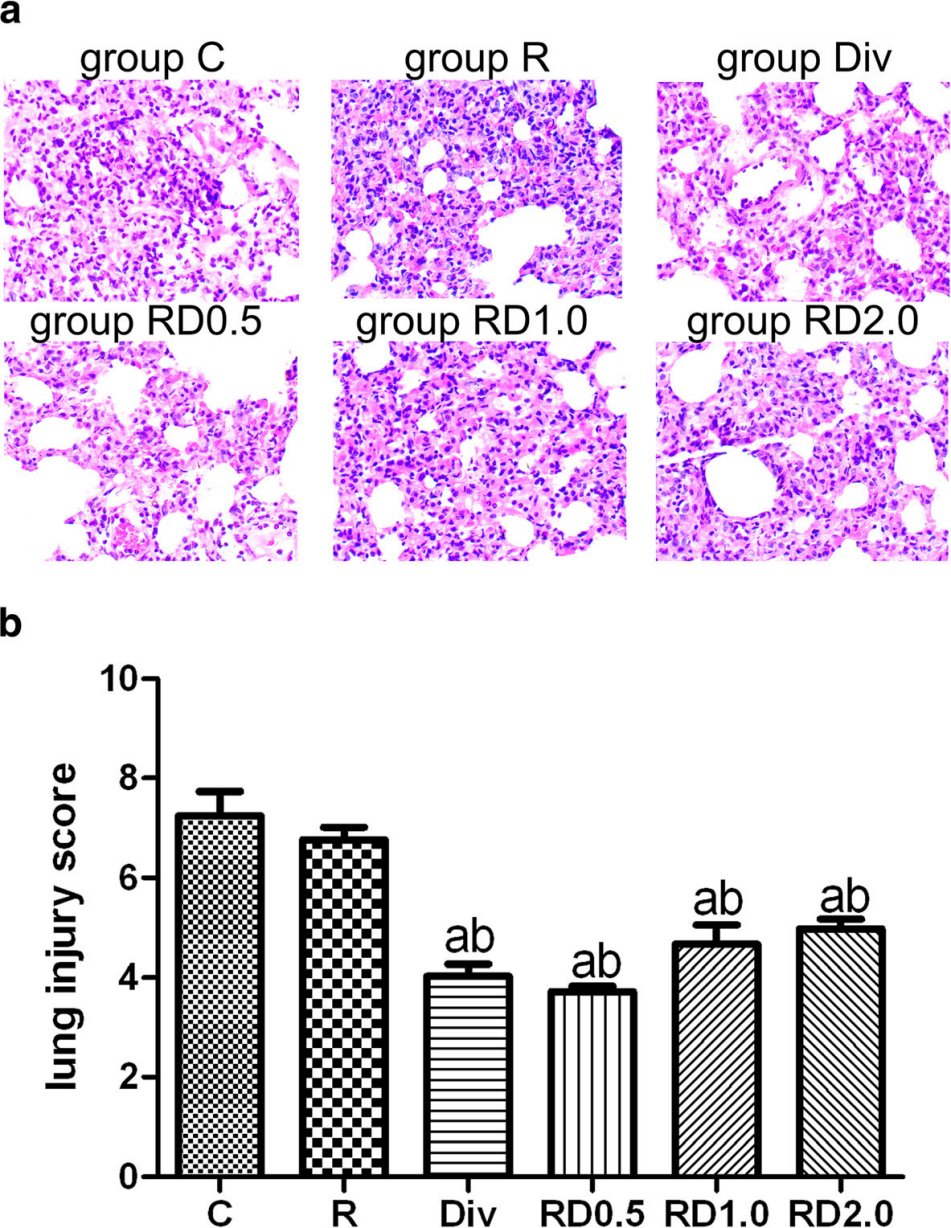
Examination of lung injury by light microscopy following one-lung ventilation. Lung injury was indicated in edema, inflammatory cell infiltration and alveolar congestion (Fig. 2a. Histological analysis revealed severe lung injury in groups C and R. Lung injury was significantly decreased in the Div, RD0.5, RD1.0, and RD2.0 groups compared with the C and R groups. No differences were found among the Div, RD0.5, RD1.0 and RD2.0 groups. Figure 2b: ^a^*P*<0.05 vs. the C group; ^b^*P*<0.05 vs. the R group. Magnification ×200. C, normal saline; R, ropivacaine; Div, intravenous DEX; RD0.5, 0.5 μg/kg DEX as an adjuvant to ropivacaine TPVB; RD1.0, 1.0 μg/kg DEX as an adjuvant to ropivacaine TPVB; RD2.0, 2.0 μg/kg DEX as an adjuvant to ropivacaine TPVB

As shown in Fig. 2, the histological evaluation showed severe lung injury in the C and R groups. No significant difference in lung injury score was observed between the C and R groups. The lung injury score was significantly lower in the Div and RD0.5 groups than in the C and R groups. However, no significant difference was observed between the Div and RD0.5 groups. Compared with the C and R groups, the lung injury score was slightly decreased in the RD1.0 and RD2.0 groups, although the differences were not significant. No significant differences were found among the Div, RD0.5, RD1.0 and RD2.0 groups.

### ELISA of cytokines

**Fig. 3:**
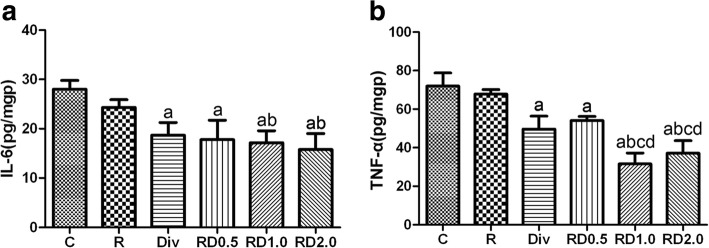
Examination of IL-6 and TNF-α by ELISA following one-lung ventilation. Figure 2a: TNF-α and IL-6 content was lower in the Div, RD0.5, RD1.0 and RD2.0 groups than in the C group; TNF-α and IL-6 content was lower in the RD1.0 and RD2.0 group than in the R group; TNF-α content was lower in the RD1.0 and RD2.0 groups than in the Div and RD0.5 groups; ^a^*P*<0.05 vs. the C group; ^b^*P*<0.05 vs. the R group; ^c^*P*<0.05 vs. the Div group; ^d^*P*<0.05 vs. the RD0.5 group. C, normal saline; R, ropivacaine; Div, intravenous DEX; RD0.5, 0.5 μg/kg DEX as an adjuvant to ropivacaine TPVB; RD1.0, 1.0 μg/kg DEX as an adjuvant to ropivacaine TPVB; RD2.0, 2.0 μg/kg DEX as an adjuvant to ropivacaine TPVB

As shown in Fig. 3, TNF-α and IL-6 content in the Div, RD0.5, RD1.0 and RD2.0 was lower than that in the C group. TNF-α and IL-6 content in the RD1.0 and RD2.0 groups was lower than that in the R group. Furthermore, TNF-α content was lower in the RD1.0 and RD2.0 groups than in the Div and RD0.5 groups. IL-6 content was slightly decreased in the RD1.0 and RD2.0 groups compared to the Div and RD0.5 groups, although the differences were not significant.

### Detection of lung cell apoptosis by the TUNEL assay

**Fig. 4:**
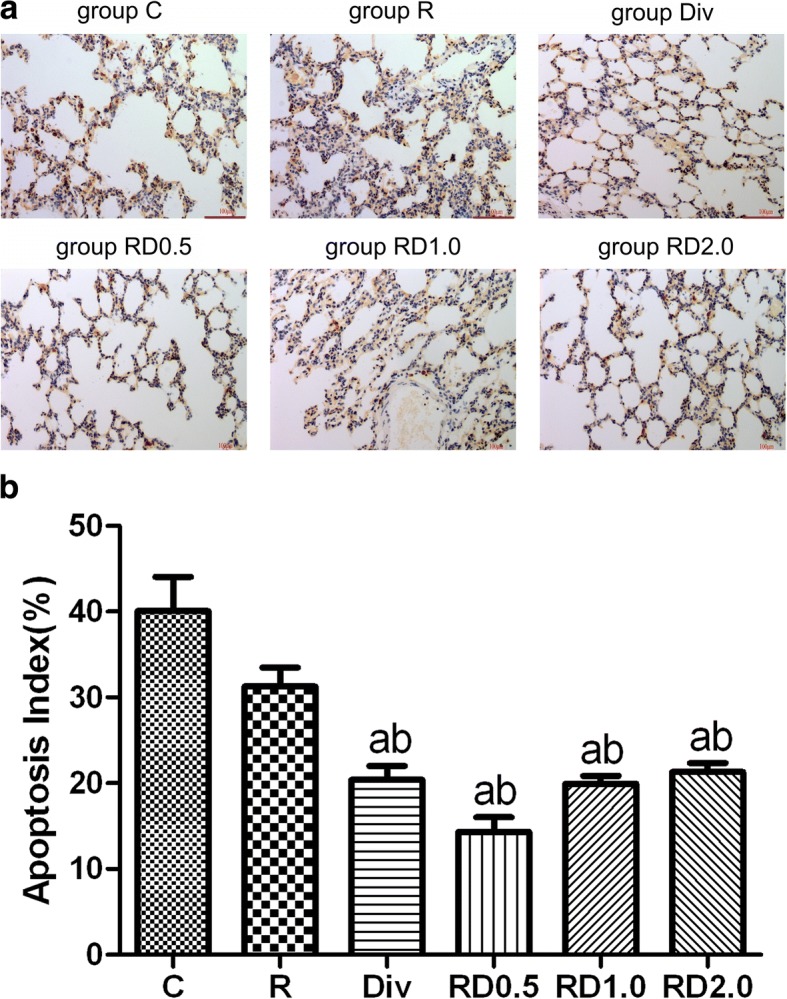
Representative images from the TUNEL apoptosis assay following one-lung ventilation. Images from the TUNEL apoptosis assay following one-lung ventilation (Fig. 4a. Yellow-brown staining indicates apoptosis. As shown in Fig. 4b the AI index was the highest in C and R groups. The AI index was significantly lower in the Div, RD0.5, RD1.0 and RD2.0 groups than in the C and R groups. Magnification × 400. C, normal saline; R, ropivacaine; Div, intravenous DEX; RD0.5, 0.5 μg/kg DEX as an adjuvant to ropivacaine TPVB; RD1.0, 1.0 μg/kg DEX as an adjuvant to ropivacaine TPVB; RD2.0, 2.0 μg/kg DEX as an adjuvant to ropivacaine TPVB

As demonstrated in Fig. 4, the AI index was the highest in groups C and R. No significant difference was observed between C group and R group. The AI index was significantly lower in the Div, RD0.5, RD1.0 and RD2.0 groups than in the C and R groups, although no significant difference was observed among the Div, RD0.5, RD1.0 and RD2.0 groups.

### Real-time PCR of miRNA-210

**Fig. 5:**
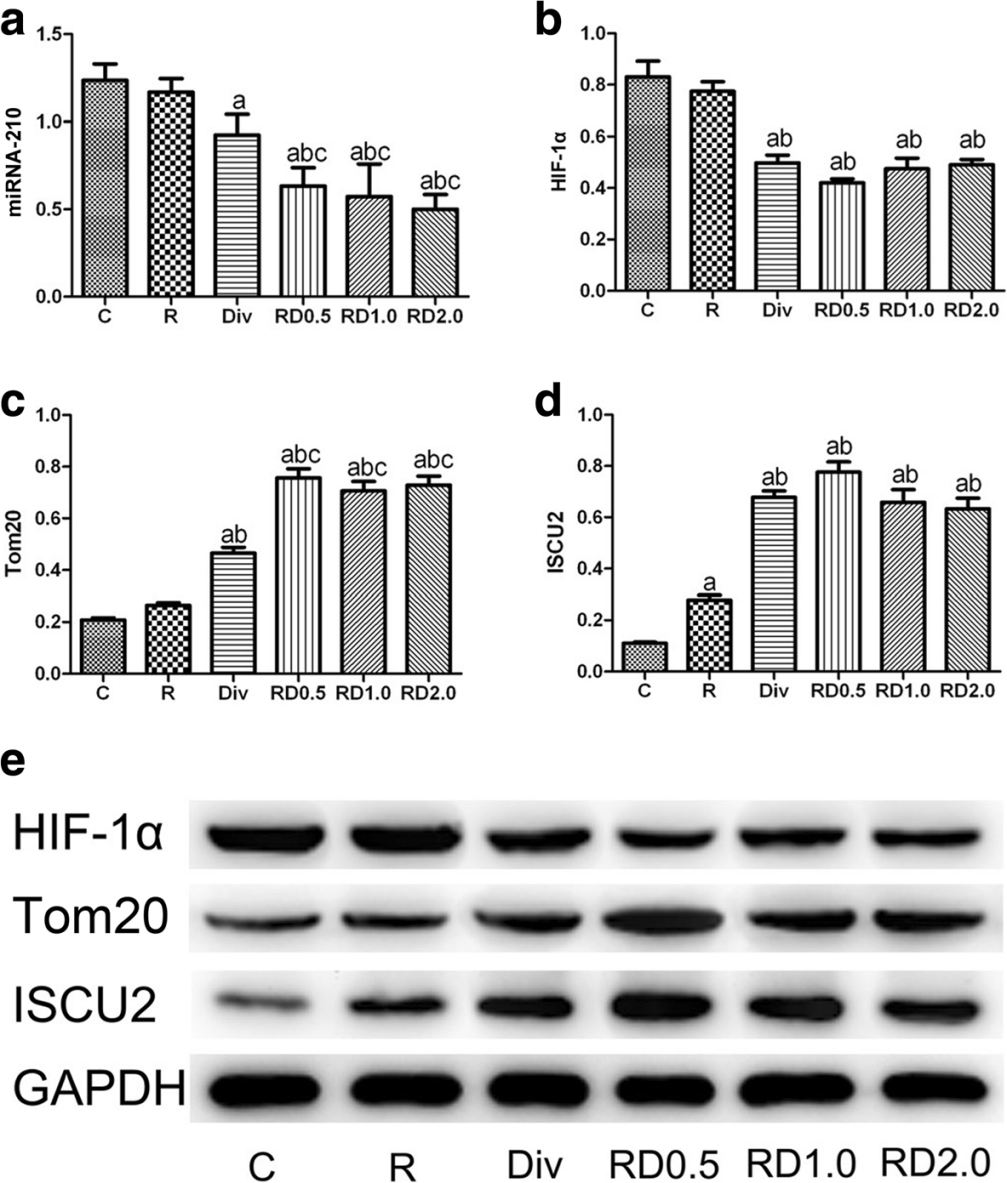
Results of real-time PCR and western blots. Real-time PCR results of miRNA-210 (**a**) and western blots of HIF-1α (**b** and **e**), Tom20 (**c** and **e**), and ISCU2 (D and E). ^a^*P*<0.05 vs. the C group; ^b^*P*<0.05 vs. the R group; ^c^*P*<0.05 vs. the Div group; ^d^*P*<0.05 vs. the RD0.5 group. C, normal saline; R, ropivacaine; Div, intravenous DEX; RD0.5, 0.5 μg/kg DEX as an adjuvant to ropivacaine TPVB; RD1.0, 1.0 μg/kg DEX as an adjuvant to ropivacaine TPVB; RD2.0, 2.0 μg/kg DEX as an adjuvant to ropivacaine TPVB

As shown in Fig. 5a, the expression of miRNA-210 in the Div, RD0.5, RD1.0 and RD2.0 groups was decreased compared with C group. The expression of miRNA-210 was lower in the RD0.5, RD1.0 and RD2.0 groups than in R group, but no significant difference was observed between the R and Div groups. The expression of miRNA-210 was lower in the RD0.5, RD1.0 and RD2.0 groups than in the Div group. No significant difference was observed among the RD0.5, RD1.0 and RD2.0 groups.

### Expression of HIF-1α, Tom20, and ISCU2 in the lung tissues by western blot

As illustrated in Fig. 5b and e, expression of HIF-1α in groups C and R were significantly increased compared with the other four groups. However, no differences in HIF-1α were observed among the Div, RD0.5, RD1.0 and RD2.0 groups.

As illustrated in Fig. 5c and e, the expression of Tom20 was significantly increased the Div, RD0.5, RD1.0 and RD2.0 groups compared with the C and R groups, and the expression of Tom20 was higher in the RD0.5, RD1.0 and RD2.0 groups than that in the Div group. No significant differences were observed among the RD0.5, RD1.0 and RD2.0 groups.

As illustrated in Fig. 5d and e, the expression of ISCU2 in C group was the lowest among all groups, and the expression of ISCU2 was higher in R group than in C group. The expression of ISCU2 was significantly higher in the Div, RD0.5, RD1.0 and RD2.0 groups than in the R group, whereas no differences in ISCU2 expression were observed among the Div, RD0.5, RD1.0 and RD2.0 groups.

## Discussion

To the best of our knowledge, the present study is the first to show that paravertebral DEX can attenuate independent lung injury during OLV, as indicated by the significant decrease in injury scores, AI, and inflammatory cytokines (TNF-α and IL-6) in the lung tissue samples. Moreover, the Div and RD0.5 groups showed no differences in lung injury. Though the administration route differed, no difference in the effect was observed. Therefore, both intravenous and paravertebral administration routes are effective methods for DEX to attenuate lung injury. To verify the dose dependency of paravertebral DEX, we included RD1.0 and RD2.0 groups, which received higher doses than the RD0.5 group. Surprisingly, no further improvements were found for these increased doses of paravertebral DEX.

As shown in Table 2, MAP and HR were significantly decreased in the Div, RD0.5, RD1.0 and RD2.0 groups than those in the C and R groups. However, patients who experienced bradycardia and hypotension in the Div group were more than those in the RD0.5 group; the occurrence of bradycardia and hypotension in the RD1.0 group was similar to those in the Div group; patients in the RD2.0 group experienced the most bradycardia and hypotension (shown in Table 1). Though these side effects were usually transient and could be easily corrected. Larger doses of paravertebral DEX induced more dose-dependent side effects. Considering patient safety, it is recommended to refrain from increasing the dose of paravertebral DEX when administered to protect against lung injury.

HIF-1 is a critical transcription factor for cell survival under hypoxic conditions [17]. HIF-1 is composed of two subunits: HIF-1α and HIF-1β. HIF-1α is the main subunit that reacts to hypoxia. Although HIF-1α primarily accumulates under hypoxic conditions, HIF-1α expression also depends on its rate of de novo synthesis. Recent studies have shown that inflammatory cytokines and other signaling molecules can also stimulate HIF-1α. Suresh’s study showed that activation of HIF-1α in type 2 cells is a major driver of acute inflammation following lung contusion [18]. Sun’s study [19] showed that HIF-1α expression can be induced and activated in rats during acute lung inflammatory damage triggered by septic lymph injection via a HIF-1α-dependent pathway. As illustrated in Fig. 5b, e, we showed that the expression of HIF-1α was upregulated in the C and R groups, which had the most severe lung injury among all groups. Lung injury was attenuated after paravertebral and intravenous DEX administration, accompanied by downregulation of HIF-1α. We believe that the upregulation of HIF-1α may be a driver of lung injury in our model. Paravertebral and intravenous administration of DEX are both effective for protecting against independent lung injury induced by OLV, and the mechanism may be related to downregulation of HIF-1α.

MicroRNAs (miRNAs) are small non-coding RNAs that are important mediators of numerous cellular processes, including the response to hypoxia [20]. In a hypoxic environment, miRNA-210 can be upregulated by HIF-1α [21]. ISCU, which encodes a scaffold protein that plays a critical role in Fe-S cluster biogenesis, is synthesized as a precursor that is located in the cytosol and then migrates to the mitochondria after mitochondrial target sequence cleavage [22]. In mammalian cells, ISCU1 is located in the cytosol, whereas ISCU2 is located in the mitochondria. ISCU2 is a key chaperone for the assembly of cellular ISCs and transportation to enzymes that are responsible for mitochondrial respiration and energy production [23]. Therefore, the suppression of ISCU2 may result in the generation of reactive oxygen species (ROS) and mitochondrial dysfunction [24, 25]. Recent studies have investigated the repression of miRNA-210 on iron-sulfur cluster (ISC) assembly proteins, and ISCU2 was predicted to contain a highly conserved binding site for miRNA-210 [26, 27]. This regulation axis, comprising HIF-1α, miRNA-210, and ISCU2, mediates the mitochondrial energy metabolism process that responds to hypoxia in different cell types. The energy metabolic shift regulated by the HIF-1α/miRNA-210/ISCU2 pathway has been revealed to participate in multiple hypoxia-related physiological and pathological processes [25, 28–30].

Mitochondria play a fundamental role in energy metabolism. Mitochondrial dysfunction can result in cellular dysfunction or initiate the cell death process. Tom20 is a mitochondrial protein marker that is translocated at the outer mitochondrial membrane. In our study, mitochondrial injury was indicated by Tom20 and ISCU2. Severe mitochondrial dysfunction significantly downregulates Tom20 and ISCU2, which mediate the mitochondrial energy metabolic process under hypoxia. As shown in Fig. 5a, c, d, e, downregulated miRNA-210 and upregulated ISCU2 and Tom20 were observed upon administration of intravenous or paravertebral DEX.

Several studies have shown that the intraoperative sedation level is also strengthened by paravertebral DEX administration. The pro-analgesia effect is achieved via peripheral action [31], but the promotion of sedation may be due to a systemic mechanism. The sedation resulting from paravertebral DEX may be caused by increased plasma concentrations through local absorption [32]. The advantage of paravertebral DEX administration is that sedation could develop both through peripheral and systemic mechanisms. This is consistent with the observation that the RD0.5 group had a similar effect as the Div group in our study. Both administration routes could protect against the lung injury induced by OLV. Based on the above analysis, the paravertebral route is recommended.

To date, there is no consensus in terms of the optimal dose of paravertebral DEX. As noted above, to determine whether or not lung injury can be further attenuated by increasing the dose of DEX, 1.0 μg/kg and 2.0 μg/kg paravertebral DEX were administered to the RD1.0 and RD2.0 groups, respectively. However, no further improvement was found in these two groups compared with the RD0.5 group. As described in a previous study and in our study, large doses of DEX have been shown to confer increased risk of sedation and bradycardia [33]. Therefore, side effects may be induced by increasing the dose of DEX. As 0.5 μg paravertebral DEX offered significant protection against lung injury, larger doses may not be advised.

The shortcomings of this study are as follows:

1. We acknowledge that this study is limited by its retrospectively registered.
2. We acknowledge type II errors will be increased after Bonferroni correction to multiple comparisons in our study, though the main negative results of our study did not conflict with clinical findings (hemodynamics, side effects, etc.). This should be a statistical limitation.
3. Based on the statistical assumption, the sample size is not enough. Though the results showed in this study is encouraging, more patients should be recruited and future trials should be implemented.
4. In our present study, fluid administration was adjusted according to the hemodynamics, blood loss and urinary output. Non-monitoring of fluid responsiveness should be a limitation. Though the intraoperative fluid balance in each arm was generally less than 500 ml and no pulmonary edema was found in any patients, 3 ml/kg/hr. as fluid maintenance may be hazardous to the lungs.
5. During OLV, in order to protect the ventilated lung, the oxygen should be reduced less than 100% unless the arterial oxygenation is not good enough. In our present study, we used 100% oxygen during OLV to all the patients, this maybe an injurious factor for the ventilated lung.
6. Continuous paravertebral blockade by catheter technique would improve intraoperative and postoperative analgesia. In our present study, we only used a single injection of DEX and ropivacaine without catheter technique. Non-use of PVB catheter technique should be a limitation.
7. Only independent lung injury was considered here; however, both lungs are injured during OLV [15].
8. Independent lung injury has two phases in OLV: collapsed injury and injury after re-inflation. For ethical reasons, harvesting lung tissue is not permitted after re-inflation. Only collapsed injury was discussed in our study, and injury after re-inflation remains unknown.
9. Whether clinical outcomes are improved by administration of paravertebral DEX requires further study.
10. We only revealed that lung injury in our model was decreased by paravertebral DEX through the HIF-1α/miRNA-210/ISCU2 axis. However, the overall mechanism, for example, how DEX affects this axis and why no difference was found between the Div and RD0.5 groups, requires more complex studies.

## Conclusions

Overall, our preliminary results showed that paravertebral dexmedetomidine as an adjuvant to ropivacaine can protect against independent lung injury during OLV. Lung injury, AI, and inflammation were alleviated by paravertebral DEX administration, which was accompanied by inhibition of the HIF-1α/miRNA-210/ISCU2 axis. In addition, 0.5 μg DEX administered both paravertebrally and intravenously can protect against independent lung injury, and inhibition of the HIF-1α/miRNA-210/ISCU2 axis may be the underlying mechanism.

## Abbreviations

ASA: American Society of Anesthesiologists
DEX: dexmedetomidine
HIF-1α: hypoxia inducible factor-1α
OLV: one-lung ventilation
TPVB: thoracic paravertebral block

## References

[1] Licker MJ, Widikker I, Robert J, Frey JG, Spiliopoulos A, Ellenberger C, Schweizer A, Tschopp JM (2006). Operative mortality and respiratory complications after lung resection for cancer: impact of chronic obstructive pulmonary disease and time trends. Ann Thorac Surg.

[2] Lohser J, Slinger P (2015). Lung injury after one-lung ventilation: a review of the pathophysiologic mechanisms affecting the ventilated and the collapsed lung. Anesth Analg.

[3] Sivrikoz MC, Tuncozgur B, Cekmen M, Bakir K, Meram I, Kocer E, Cengiz B, Elbeyli L (2002). The role of tissue reperfusion in the reexpansion injury of the lungs. Eur J Cardiothorac Surg.

[4] Tekinbas C, Ulusoy H, Yulug E, Erol MM, Alver A, Yenilmez E, Geze S, Topbas M (2007). One-lung ventilation: for how long?. J Thorac Cardiovasc Surg.

[5] Lee SH, Kim N, Lee CY, Ban MG, Oh YJ (2016). Effects of dexmedetomidine on oxygenation and lung mechanics in patients with moderate chronic obstructive pulmonary disease undergoing lung cancer surgery: a randomised double-blinded trial. Eur J Anaesthesiol.

[6] Xia R, Xu J, Yin H, Wu H, Xia Z, Zhou D, Xia ZY, Zhang L, Li H, Xiao X (2015). Intravenous infusion of Dexmedetomidine combined isoflurane inhalation reduces oxidative stress and potentiates hypoxia pulmonary vasoconstriction during one-lung ventilation in patients. Mediat Inflamm.

[7] Gao S, Wang Y, Zhao J, Su A (2015). Effects of dexmedetomidine pretreatment on heme oxygenase-1 expression and oxidative stress during one-lung ventilation. Int J Clin Exp Pathol.

[8] Krediet AC, Moayeri N, van Geffen GJ, Bruhn J, Renes S, Bigeleisen PE, Groen GJ (2015). Different approaches to ultrasound-guided thoracic paravertebral block: an illustrated review. Anesthesiology.

[9] Mohamed SA, Fares KM, Mohamed AA, Alieldin NH (2014). Dexmedetomidine as an adjunctive analgesic with bupivacaine in paravertebral analgesia for breast cancer surgery. Pain Physician.

[10] Dutta V, Kumar B, Jayant A, Mishra AK (2017). Effect of continuous paravertebral Dexmedetomidine administration on intraoperative anesthetic drug requirement and post-thoracotomy pain syndrome after thoracotomy: a randomized controlled trial. J Cardiothorac Vasc Anesth.

[11] Marhofer P, Brummett CM (2016). Safety and efficiency of dexmedetomidine as adjuvant to local anesthetics. Curr Opin Anaesthesiol.

[12] Packiasabapathy SK, Kashyap L, Arora MK, Batra RK, Mohan VK, Prasad G, Yadav CS (2017). Effect of dexmedetomidine as an adjuvant to bupivacaine in femoral nerve block for perioperative analgesia in patients undergoing total knee replacement arthroplasty: a dose-response study. Saudi J Anaesth.

[13] Xu J, Yang X, Hu X, Chen X, Zhang J, Wang Y (2018). Multilevel thoracic paravertebral block using Ropivacaine with/without Dexmedetomidine in video-assisted Thoracoscopic surgery. J Cardiothorac Vasc Anesth.

[14] Hassan ME, Mahran E (2017). Evaluation of the role of dexmedetomidine in improvement of the analgesic profile of thoracic paravertebral block in thoracic surgeries: a randomised prospective clinical trial. Indian J Anaesth.

[15] Kozian A, Schilling T, Freden F, Maripuu E, Rocken C, Strang C, Hachenberg T, Hedenstierna G (2008). One-lung ventilation induces hyperperfusion and alveolar damage in the ventilated lung: an experimental study. Br J Anaesth.

[16] Zhang W, Zhang JQ, Meng FM, Xue FS (2016). Dexmedetomidine protects against lung ischemia-reperfusion injury by the PI3K/Akt/HIF-1alpha signaling pathway. J Anesth.

[17] Semenza GL (2001). HIF-1 and mechanisms of hypoxia sensing. Curr Opin Cell Biol.

[18] Suresh MV, Ramakrishnan SK, Thomas B, Machado-Aranda D, Bi Y, Talarico N, Anderson E, Yatrik SM, Raghavendran K (2014). Activation of hypoxia-inducible factor-1alpha in type 2 alveolar epithelial cell is a major driver of acute inflammation following lung contusion. Crit Care Med.

[19] Sun HD, Liu YJ, Chen J, Chen MY, Ouyang B, Guan XD (2017). The pivotal role of HIF-1alpha in lung inflammatory injury induced by septic mesenteric lymph. Biomed Pharmacother.

[20] Bhaskaran M, Mohan M (2014). MicroRNAs: history, biogenesis, and their evolving role in animal development and disease. Vet Pathol.

[21] Majmundar AJ, Wong WJ, Simon MC (2010). Hypoxia-inducible factors and the response to hypoxic stress. Mol Cell.

[22] Kilpivaara O, Aaltonen LA (2013). Diagnostic cancer genome sequencing and the contribution of germline variants. Science.

[23] Tong WH, Rouault TA (2006). Functions of mitochondrial ISCU and cytosolic ISCU in mammalian iron-sulfur cluster biogenesis and iron homeostasis. Cell Metab.

[24] He MD, Xu SC, Lu YH, Li L, Zhong M, Zhang YW, Wang Y, Li M, Yang J, Zhang GB (2011). L-carnitine protects against nickel-induced neurotoxicity by maintaining mitochondrial function in neuro-2a cells. Toxicol Appl Pharmacol.

[25] He M, Lu Y, Xu S, Mao L, Zhang L, Duan W, Liu C, Pi H, Zhang Y, Zhong M (2014). MiRNA-210 modulates a nickel-induced cellular energy metabolism shift by repressing the iron-sulfur cluster assembly proteins ISCU1/2 in neuro-2a cells. Cell Death Dis.

[26] Chan SY, Zhang YY, Hemann C, Mahoney CE, Zweier JL, Loscalzo J (2009). MicroRNA-210 controls mitochondrial metabolism during hypoxia by repressing the iron-sulfur cluster assembly proteins ISCU1/2. Cell Metab.

[27] Lee DC, Romero R, Kim JS, Tarca AL, Montenegro D, Pineles BL, Kim E, Lee J, Kim SY, Draghici S (2011). miR-210 targets iron-sulfur cluster scaffold homologue in human trophoblast cell lines:siderosis of interstitial trophoblasts as a novel pathology of preterm preeclampsia and small-for-gestational-age pregnancies. Am J Pathol.

[28] Chen Z, Li Y, Zhang H, Huang P, Luthra R (2010). Hypoxia-regulated microRNA-210 modulates mitochondrial function and decreases ISCU and COX10 expression. Oncogene.

[29] Favaro E, Ramachandran A, McCormick R, Gee H, Blancher C, Crosby M, Devlin C, Blick C, Buffa F, Li JL (2010). MicroRNA-210 regulates mitochondrial free radical response to hypoxia and Krebs cycle in cancer cells by targeting iron sulfur cluster protein ISCU. PLoS One.

[30] Muralimanoharan S, Maloyan A, Mele J, Guo C, Myatt LG, Myatt L (2012). MIR-210 modulates mitochondrial respiration in placenta with preeclampsia. Placenta.

[31] Konakci S, Adanir T, Yilmaz G, Rezanko T (2008). The efficacy and neurotoxicity of dexmedetomidine administered via the epidural route. Eur J Anaesthesiol.

[32] Zhang X, Wang D, Shi M, Luo Y (2017). Efficacy and safety of Dexmedetomidine as an adjuvant in epidural analgesia and anesthesia: a systematic review and meta-analysis of randomized controlled trials. Clin Drug Investig.

[33] Zeng X, Jiang J, Yang L, Ding W (2017). Epidural Dexmedetomidine reduces the requirement of Propofol during Total intravenous Anaesthesia and improves analgesia after surgery in patients undergoing open thoracic surgery. Sci Rep.

